# Successful Operation for the Relief of Hereditary Cataract

**Published:** 1852-12

**Authors:** J. Richardson

**Affiliations:** Springfield


ARTICLE III.—*Successful Operation for the relief of Hereditary Cataract, by* J. RICHARDSON, M. D.

MARGARET HANNAH, a native of Ireland, called on me in the Fall of 1849, suffering from congenital cataract of both eyes. She was twenty-four years of age. There was complete opacity of the enses of both eyes; consequently, total blindness. After dilating the pupil by the application of belladonna, I operated on the left eye by breaking up the lens with the couching needle, and depressing it below the axis of vision, where I retained it until it became imbedded in the vitreous humor; then I had the patient placed in a dark room, and re-applied belladonna from time to time, and directed the frequent application of tepid water to the eye.— In four weeks from the time of the operation, she could bear a considerable degree of light, and began to distinguish objects clearly. I then operated on the right eye, with like success; though it was followed by more inflammation than succeeded the first operation, and a portion of the lens escaped into the anterior chamber, which, however, soon became absorbed by a moderate ptyalism.— It is now near three years since the operations were performed.— She has attended school for the last two years—has learned to read well, and write neatly. The greatest difficulty she has had to encounter, since her introduction into the world of light, was to learn the relative difference in the size, distances, and colors of objects. For a considerable period of time, she could not distinguish red from yellow, or blue from green, with certainty. These difficulties she has overcome, however, and she now enjoys as good sight as any person could well have, minus the lens; this deficiency is, in her case, in a great measure, obviated by the use of lenticular glasses. The mother of the patient lost her sight at the age of ten years, by small pox. After her marriage, she became the mother of three children, two of whom were born blind; so that Miss Hannah's blindness was not only congenital, but hereditary.

Springfield, October 25, 1852.

